# Delivery of Nerve Growth Factor *via* Exosome Attenuates Neuropathic Pain in a Rat Model of Chronic Constriction Injury

**DOI:** 10.2174/011570159X352871250404101246

**Published:** 2025-10-08

**Authors:** Yangyi Li, Chengbo Chen, Changsheng Su, Shunyuan Li, Zhibin Wen, Yifan Zheng

**Affiliations:** 1 Department of Anesthesiology, Quanzhou First Hospital Affiliated to Fujian Medical University, No. 1028 South of Anji Road, Quanzhou, 362000, Fujian, China;; 2 Department of Cardiology, Quanzhou First Hospital Affiliated to Fujian Medical University, No. 1028 South of Anji Road, Quanzhou, 362000, Fujian, China

**Keywords:** Neuropathic pain, exosome, neurotrophic factor, inflammation, CCI rats, von Frey test, protein levels

## Abstract

**Introduction:**

The nerve growth factor (NGF) is a crucial neurotrophic factor with the ability to induce neuronal differentiation. However, whether NGF-loaded exosomes (Exo-NGF) can alleviate neuropathic pain in chronic constriction injury (CCI) rats remains unclear.

**Methods:**

A neuropathic pain model was established using CCI rats. The pain was assessed using the von Frey test and the hot plate test. Exo-NGF was collected from HEK293 cells transfected with an NGF plasmid. The diameter of Exo-NGF was determined using transmission electron microscopy. Protein levels of inflammatory factors, including IL-18, IL-1β, and TNF-α, were measured using enzyme-linked immunosorbent assay, and their mRNA levels were evaluated using qPCR. The NOD-like receptor family pyrin domain-containing protein 3 (NLRP3) protein levels were determined using immunostaining and Western blot.

**Results:**

NGF protein and mRNA were highly expressed in Exo-NGF. The mRNA in Exo-NGF was successfully delivered into neural stem cells and promoted their differentiation. Injection of Exo-NGF into the spinal dorsal horn significantly alleviated mechanical allodynia and thermal hyperalgesia. Additionally, Exo-NGF reduced levels of IL-18, IL-1β, and TNF-α. NLRP3 and its key components, including apoptosis-associated speck-like protein and caspase-1, were also reduced by Exo-NGF treatment in CCI rats.

**Discussion:**

Our findings highlight the therapeutic potential of exosome-based NGF delivery for treating chronic pain conditions.

**Conclusion:**

Exo-NGF significantly alleviates neuropathic pain by suppressing inflammation and NLRP3 activation.

## 1 INTRODUCTION

The somatosensory nervous system can be damaged or injured, leading to an abnormal sensation of pain known as neuropathic pain, which is characterized by symptoms of allodynia and hyperalgesia [1]. Neuropathic pain significantly impacts a patient's quality of life and places a burden on society. It is estimated that up to 7% to 10% of the global population suffers from neuropathic pain, making it a major clinical challenge [2]. Understanding the underlying mechanisms is crucial for developing effective treatments for neuropathic pain.

The primary pathological mechanisms of neuropathic pain are intricately linked to central and peripheral nerve sensitization, inflammation, oxidative stress, and the release of proinflammatory cytokines [3-6]. Previous research has highlighted the role of neuroinflammation in the development of neuropathic pain by enhancing synaptic efficacy and increasing neuronal excitability in nociceptive pathways [7]. These findings have been consistently validated in the neuronal circuits of the spinal dorsal horn in patients with neuropathic pain [8].

The NOD-like receptor family pyrin domain-containing protein 3 (NLRP3) inflammasome is a critical molecular platform that can be activated by harmful signals [9]. The NLRP3 inflammasome consists of the pattern recognition receptor NLRP3, the adaptor protein apoptosis-associated speck-like protein (ASC), and the procaspase-1 enzyme [10]. Upon activation, the inflammasome triggers pyroptosis, a newly identified form of pro-inflammatory programmed cell death [11]. This process results in the rupture of the cell membrane and the release of cellular contents, initiating inflammatory cascades. It has been reported that NLRP3 activation leads to the release of IL-18 and IL-1β [12, 13]. Multiple studies have suggested that activation of the NLRP3 inflammasome is involved in the development of neuropathic pain [14, 15].

Exosomes, ranging from 30 to 200 nm in diameter, are small single-membrane vesicles containing abundant proteins, lipids, nucleic acids, and glycoconjugates [16]. Due to their ability to efficiently traverse the blood-brain barrier and deliver therapeutic molecules specifically to the central nervous system, exosomes are promising biomarkers for the treatment of neurological disorders [17].

The nerve growth factor (NGF) is a crucial neurotrophic factor that plays an essential role in regulating neuronal survival, neurotransmitter release, and synaptic function [18]. Elevating NGF has been shown to effectively mitigate histological injury, prevent neuron loss and apoptosis, and promote nerve regeneration in injured rat spinal cords by facilitating the differentiation of neural stem cells (NSCs) into functional neurons [19, 20]. Based on the known biological functions of NGF, it is reasonable to hypothesize that NGF-overexpressing exosomes may positively impact the repair of injured spinal cord tissues. Therefore, we established a chronic constriction injury (CCI) model to explore the function of NGF-overexpressing exosomes on neuropathic pain and to elucidate the mechanism by which NGF alleviates neuropathic pain.

## 2 MATERIALS AND METHODS

### 2.1 CCI Model

A total of 48 male Sprague-Dawley rats at 3 months old, weighing 250-280 g, were purchased from Cyagen Biosciences (Suzhou, China) and housed in an SPF facility (3 rats per cage) with free access to water and food. The rats were randomly allocated to the groups. The CCI model was performed on the rats to induce neuropathic pain following a previously established method [21]. To avoid the potential interference of hormonal fluctuations in female rats on behavioral tests, only male rats were used in the current study. The rats were anesthetized with intraperitoneal injections of a xylazine-ketamine mixture. An incision was made on the lateral surface of the left thigh to access the biceps femoris and gluteus muscles. After separating these muscles through blunt dissection, the sciatic nerve was exposed and gently retracted. Using a nonabsorbent 5-0 silk suture, three loose ligations were applied to the dorsal third to half of the common sciatic nerve at the upper-thigh level. In the CCI group, isolated sciatic nerves in rats were ligated by threads. In the sham group, sciatic nerves were isolated without ligation. The neuropathic behaviors observed in the CCI model rats were directly attributed to nerve damage induced by CCI modeling. Pain sensitivity and heat sensitivity detection are classic methods used in behavioral testing [22-24]. Mice have been euthanized by CO_2_ inhalation after the experiments. All animal experiments have been approved by Quanzhou First Hospital Affiliated to Fujian Medical University (#2023-DW-37).

### 2.2 Cell Culture

HEK293 cells (ATCC) were cultured in Dulbecco’s Modified Eagle’s Medium (DMEM; Gibco, Grand Island, NY) supplemented with 10% fetal bovine serum (FBS; Gibco) and 1% penicillin-streptomycin (Gibco). NSCs (Thermo, MA, USA) were cultured in DMEM/F-12 (Gibco) supplemented with 2% B27 (Thermo), 1% N2 (Thermo), 20 ng/mL epidermal growth factor (Thermo), 20 ng/mL basic fibroblast growth factor (Thermo) and 1% penicillin-streptomycin. All cells were maintained in an incubator with 5% CO_2_ at 37^o^C.

### 2.3 Transfection of NGF Plasmid

The NGF-overexpressing plasmid was purchased from a commercial company (Genewiz, Suzhou, China). HEK293 cells were cultured in a 10-cm dish. When cell confluence reached 80%, transfection was performed as follows: the plasmid was diluted in Opti-MEM (Thermo), followed by the addition of an appropriate amount of PolyJet reagent (SignaGen). The DNA/PolyJet complex was incubated at room temperature for 15 minutes, then the mixture was added dropwise into the dish.

### 2.4 Exosome Preparation

Exosomes were isolated from the supernatant of HEK293 cells transfected with the NGF plasmid. Before collecting the culture medium, HEK293 cells were washed twice with PBS and then cultured in fresh medium supplemented with 10% exosome-depleted FBS. Following 48 hours of incubation, the supernatant was subjected to sequential ultracentrifugation at 2,000 g for 30 minutes, 10,000 g for 30 minutes, and 100,000 g for 2 hours at 4^o^C. The exosomes were subsequently washed with PBS and resuspended for further characterization.

### 2.5 The Injection of Exosome

The intrathecal injection of HEK293-NGF-derived exosomes was performed according to a previously described method [25]. A micro-syringe was inserted vertically into the skin after identifying the puncture site between the L5 and L6 spinous processes. Once the spinal column was reached, the syringe angle was adjusted to 30 degrees and then carefully inserted into the intervertebral space. The puncture was confirmed by the characteristic “S” shape of the tail. Exosomes (5 μg in 25 μL) were injected on days 2, 4, and 6 following CCI surgery. Rats in the sham and CCI groups received an equal volume of 0.9% saline as controls.

### 2.6 Pain Behavioral Quantification

The Von Frey test was conducted to evaluate the response to a normally harmless stimulus. An electroVon Frey (e-VF; Ugo Basile Biological Instruments, model 37000-007) was used to measure the movement threshold of the right hind paw. The animals were placed in plastic observation chambers with a mesh floor and given 10 minutes to acclimate. The movement threshold was determined as the average force required to withdraw the paw across three trials.

The thermal threshold in rats was assessed as follows: Rats were placed on a hot plate (Ugo Basile Biological Instruments, model 35150-001) set at 52°C, and the response time (in seconds) was recorded. The animals were acclimated for 10 minutes in plastic observation chambers before being transferred to the 52°C apparatus. Responses such as pawing, licking, jumping, and taking two consecutive steps were measured. To prevent tissue damage, a 10-minute interval between trials was maintained, and the cutoff time at 52°C was 20 seconds. The thermal threshold was determined by the average response time across three trials. Tests for thermal hyperalgesia and mechanical allodynia were performed before and after injury on days 3, 7, 14, and 21. Tests for thermal hyperalgesia and mechanical allodynia were performed before the injury and on days 3, 7, 14, and 21 post-injury, according to previous studies [22, 23].

### 2.7 qPCR

The total RNA was extracted using trizol (Invitrogen, Waltham, MA) and reversed into cDNA using a cDNA synthesis kit (Yeason, Shanghai, China). Consequently, the relative expression of targeted genes was quantified using a qPCR mix. The primers of target genes were as follows: forward primer of *NGF*, 5’-AGCGGCGATAGCTGCACG-CGTGGCG-3’; the reverse of *NGF*, 5’-CAGATCCTGA-GTGTCTGCAGCTTCAC-3’. Forward primer of *GAPDH*, 5’-GAAGGCTGGGGCTCATTT-3’; reverse primer of *GAPDH*, 5’-CAGGAGGCATTGCTGATGAT-3’. Forward primer of *NLRP3*, GGTGACCITGTGTGIGCTG; reverse primer of *NLRP3*, ATGTCCTGAGCCATGGAAGC. Forward of *Caspase1*, GACCGAGTGGTICCCTCAAG; reverse of *Caspase1*, GACGTGTACGAGTGGGTGT. Forward primer of *ASC*, GGACAGTACCNGGCAGTTCG; reverse primer of *ASC*, GTCACCAGTAGGGCTGTGT. Forward primer of *IL-1β*, GGGATGATGACGACCTGC; reverse primer of *IL-1β*, CCACTIGTTGGCTTATGTT. Forward primer of IL-18, CAACCGCAGTAATACGGAGC; reverse primer of *IL-18*, TCTGGTCTGGGATTCGTTGG. Forward primer of *TNF-α*, TGATCGGTCCCAACAAGGA; reverse primer of *TNF-α*, TGCTGGTGGIIGCTACGA. The *GAPDH* was loaded as the internal control.

### 2.8 Western Blot

Total protein from the spinal dorsal horn on the lesioned side was lysed using RIPA buffer. Protein concentration was determined using a BCA kit (Yeason, Shanghai, China). Approximately 20 μg of denatured protein was loaded onto an SDS-PAGE gel. The resolved proteins were transferred onto a PVDF membrane and blocked at room temperature for 1 hour using 5% non-fat milk. The PVDF membrane was then incubated overnight at 4°C with the following primary antibodies: NLRP3 (ab283819, Abcam, MA, USA), Tsg101 (72312, Cell Signaling, MA, USA), Alix (92880, Cell Signaling), CD63 (67605, Proteintech, Wuhan, China), NGF (abs156060, Absin, Beijing, China), Tuj-1 (5568, Cell Signaling), MAP-2 (4542, Cell Signaling), ASC (67494, Cell Signaling), pro-caspase1 (ab179515, Abcam), caspase1 (MCE, Shanghai, China) and GAPDH (60004, Proteintech). After three washes, the PVDF membrane was incubated with a secondary antibody at room temperature for 1 hour. Following additional washes, protein detection was performed using an ECL reagent (Millipore, MA, USA). Relative protein intensity was quantified using ImageJ.

### 2.9 Immunostaining

The sections were deparaffinized in xylene and then rehydrated in ethanol. Antigen retrieval was performed in pepsin solution (GBI Labs, #E06-50) for 10 minutes. The sections were then blocked with 5% bovine serum albumin (BSA; Sigma, A7030) for 1 hour at room temperature. Next, the sections were incubated overnight at 4°C with a primary antibody against NLRP3 (68102, Proteintech). After three washes with PBS-T, the sections were incubated with diluted donkey anti-rabbit Alexa 488 (712-545, Jackson Immunoresearch, PA, USA) for 1 hour at room temperature. Following three additional washes with PBS-T, the slides were counterstained with 4′,6-diamidino-2-phenylindole (DAPI). After washing, cover glasses were mounted using a water-soluble fluorescent mounting medium (Dako Fluorescence Mounting Medium; Agilent, Santa Clara, #S3023). Immunofluorescence analysis was conducted using a digital slide scanner (Zeiss Axio Scan Z1, Carl Zeiss), and the regions of interest (ROIs) were selected at random.

### 2.10 ELISA

ELISA kits for detecting IL-18 (ab281268, Abcam), IL-1β (ab255731, Abcam), and TNF-α (ab236712, Abcam) were purchased from Abcam. Samples were lysed and measured using a standard curve, following the manufacturer’s instructions.

### 2.11 Statistics Analysis

All the statistical data were presented as mean/standard deviation (SD) and analyzed with GraphPad software. The significance of the comparison was assessed using an unpaired t-test with Welch's correction, two-way repeated measures, or Brown-Forsythe ANOVA test, followed by Dunnett's T3 multiple comparisons test.

## 3 RESULTS

### 3.1 Characterization of NGF Overexpressed Exosomes (Exo-NGF)

**Fig. (1):**
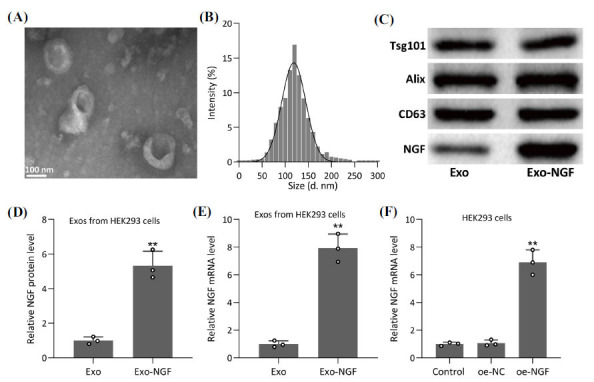
Characterization of NGF overexpressed exosomes. (**A**) TEM image of exosomes derived from NGF overexpressed HEK293 cells. (**B**) Size distribution of the exosomes derived from NGF overexpressed HEK293 cells obtained through Nanoparticle Tracking Analysis. (**C**) Detection of Tsg101, Alix, CD63, and NGF expressions by Western blot analysis from exosomes derived from HEK293 cells or NGF overexpressed HEK293 cells. (**D**) Comparison of the NGF protein levels between exosomes derived from HEK293 cells or NGF overexpressed HEK293 cells. CD63 was used as a loading control, and the expression was normalized to control (exosomes derived from HEK293 cells). (**E**) Comparison of the NGF mRNA level between exosomes derived from HEK293 cells or NGF overexpressed HEK293 cells. (**F**) Comparison of the NGF mRNA level from HEK293 cells or NGF overexpressed HEK293 cells. Data were presented as mean ± SD. **p < 0.01 compared to control. Unpaired t-test with Welch's correction or Brown-Forsythe ANOVA test followed by Dunnett's T3 multiple comparisons test.

The secreted exosomes from the HEK293 cells transfected with NGF plasmid were isolated by sequential ultracentrifugation. The images of Exo-NGF were scanned using a transmission electron microscope (TEM), as shown in Fig. (**1A**). The average size of exosomes was about 120 nm in diameter (Fig. **1B**). Afterward, the exosomal markers, including CD63, Alix, and Tsg101, were detected using Western blot in exosomes from HEK293 cells and NGF-HEK293 cells. The result showed that the exosomes from HEK293 and NGF-HEK293 cells expressed exosomal markers, demonstrating that the exosomes were successfully extracted from HEK293 cells (Fig. **1C**). To study whether NGF was loaded in exosomes from NGF-HEK293 cells, the protein and mRNA levels of NGF were detected by Western blot and qPCR in exosomes. The result unveiled that the protein level (Figs. **1C**, **1D**, **F** (DFn, Dfd) = 18.82 (2, 2)) and mRNA level (Figs. **1E**, **F** (DFn, Dfd) = 15.61 (2, 2)) of NGF were dramatically higher in exosomes from NGF-HEK293 cells than HEK293 cells. Simultaneously, the mRNA level of NGF was also higher in NGF-overexpressed HEK293 cells compared to HEK293 cells or vector-transfected HEK293 cells (Fig. **1F**, F* (DFn, DFd) = 117.0 (2.000, 2.328)). These findings proved that NGF was successfully expressed in exosomes from NGF-HEK293 cells.

### 3.2 Exo-NGF Effectively Delivers NGF into Recipient Cells

**Fig. (2):**
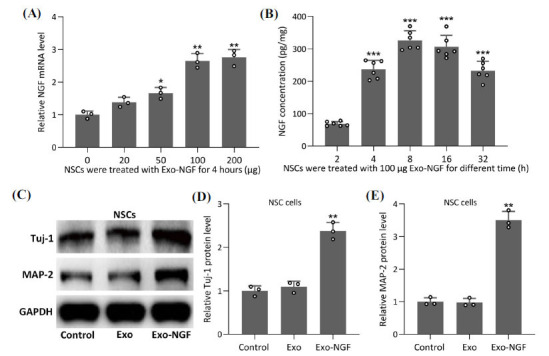
NGF overexpressed exosomes effectively delivered NGF into NSCs and promoted differentiation of NSCs into neurons. (**A**) The level of NGF mRNA in NSCs treated with different doses of NGF overexpressed exosomes. NSCs were harvested at 4 h. (**B**) The level of NGF protein in NSCs cells treated with 100 μg Exo-NGF. NSCs were harvested at 2, 4, 8, 16, and 32 h. (**C**) NSCs were treated with 100 μg exosomes derived from HEK293 cells (Exo) or NGF overexpressed HEK293 cells (Exo-NGF) and then culture for 2 weeks, Western blot was used to measure the protein level of Tuj-1 and MAP-2. GAPDH was used as a loading control and the expressions of Tuj-1 (**D**) and MAP-2 (**E**) were normalized to control. Data were presented as mean ± SD. *p < 0.05, **p < 0.01, ***p < 0.001 compared to control. Brown-Forsythe ANOVA test followed by Dunnett's T3 multiple comparisons test.

Based on our findings, Exo-NGF has been shown to contain abundant NGF mRNA and protein. It is critical to study whether Exo-NGF can deliver NGF into recipient cells after exosomes are endocytosed by recipient cells. Afterwards, NSCs were incubated with 0, 20, 50, 100, 200, or 300 μg Exo-NGF for 4 h. Subsequently, the NGF mRNA in NSCs was detected using qPCR and found to gradually be elevated with the increase of exosomes amount (Fig. **2A**, F* (DFn, DFd) = 52.12 (4.000, 8.082)). To assess whether the delivered NGF mRNA could be translated into protein in recipient cells, the NSCs were incubated with 100 μg Exo-NGF for 4 h. Consequently, the medium was refreshed, and NSCs were cultured for an additional 2, 4, 8, 16, and 32 h. The result revealed that the NGF protein level peaked at 8 h, followed by a slow decrease from 16 to 32 h (Fig. **2B**, F* (DFn, DFd) = 80.43 (4.000, 19.69)). This finding indicated that the delivered mRNA could be translated into NGF protein in NSCs in a time-dependent manner.

To evaluate the effect of NGF protein on NSCs, the specific markers of neuron cells, including Tuj-1 and microtubule-associated protein-2 (MAP-2), were detected. The Tuj-1 and MAP-2 levels were highest in NSCs treated with Exo-NGF compared to NSCs or NSCs treated with exosomes (Figs. **2C**-**E**; F* (DFn, DFd) = 79.41 (2.000, 4.781) and F* (DFn, DFd) = 182.1 (2.000, 3.697)). Taken together, it was concluded that Exo-NGF could promote neuron cell differentiation.

### 3.3 Exo-NGF Attenuated Mechanical Allodynia and Thermal Hyperalgesia in CCI Rats

**Fig. (3):**
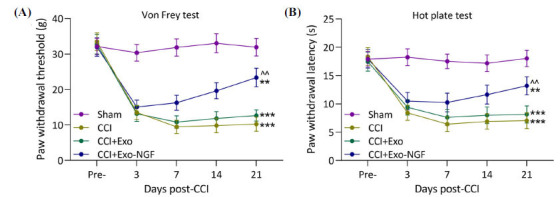
NGF overexpressed exosomes attenuated mechanical allodynia (**A**) and thermal hyperalgesia (**B**) in CCI rats. n = 6 for each group. Data were presented as mean ± SD. **p < 0.01, ***p < 0.001 compared to Sham. ^^*p* < 0.01 compared to the CCI group. Two-way repeated measures ANOVA test followed by Dunnett's T3 multiple comparisons test.

Prior to the Exo-NGF treatment, it was verified that there were no significant variances in the baseline of mechanical allodynia and thermal hyperalgesia among the sham, CCI, CCI+ Exo, and CCI+ Exo-NGF groups in the CCI rat model (Figs. **3A**, **B**). To evaluate the antinociceptive effect of Exo-NGF on the alleviation of neuropathic pain, Exo-NGF was intrathecally injected into the model rats on days 2, 4, and 6 after CCI surgery. As expected, Exo-NGF treatment significantly relieved CCI-induced mechanical allodynia and thermal hyperalgesia on days 7 post-CCI (Figs. **3A**, **B**, F* (DFn, DFd): F* (12, 140) = 38.91 and F* (12, 140) = 18.80). Exo-NGF showed increased effectiveness in reducing mechanical allodynia and thermal hyperalgesia over time (Figs. **3A**, **B**). Of note, Exo-NGF treatment failed to significantly affect the neuropathic pain behaviors of normal mice (Figs. **S1A** and **S1B**).

### 3.4 NGF Overexpressed Exosomes Attenuated Inflammatory Factor Secretion

**Fig. (4):**
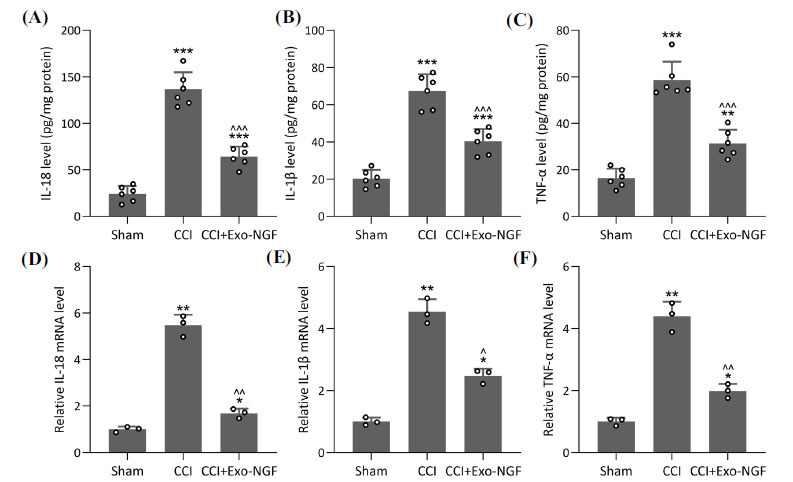
NGF overexpressed exosomes attenuated inflammatory factor secretion in CCI rats. The levels of IL-18 (**A**), IL-1β (**B**), and TNF-α (**C**) in the spinal dorsal horn of CCI rats were tested by ELISA. n = 6 for each group. The mRNA expressions of IL-18 (**D**), IL-1β (**E**), and TNF-α (**F**) in the spinal dorsal horn of CCI rats were measured by RT-qPCR. n = 3 repeats for each group (6 tissue homogenates were mixed for each group). Data were presented as mean ± SD. *p < 0.05, **p < 0.01, ***p < 0.001 compared to Sham. ^*p* < 0.05, ^^*p* < 0.01, ^^^*p* < 0.001 compared to CCI group. Brown-Forsythe ANOVA test followed by Dunnett's T3 multiple comparisons test.

To investigate the effect of Exo-NFG on the alleviation of the inflammatory response, the protein levels and mRNA levels of inflammatory factors were detected by ELISA and qPCR. IL-18, IL-1β, and TNF-α are three specific inflammatory factors that are representative of typical inflammatory responses. Especially, IL-18 and IL-1β are associated with the NLRP3 inflammasome [26]. The analysis of these three factors showed that the level of IL-18, IL-1β, and TNF-α was higher in CCI rats compared to that in control rats (Figs. **4A**-**C**). When the CCI rats were treated with Exo-NGF, the level of these three inflammatory factors significantly decreased (Figs. **4A**-**C**, F* (DFn, DFd) = 68.73 (2.000, 11.98), 111.8 (2.000, 10.33) and 72.06 (2.000, 12.00)). Afterwards, the mRNA level of IL-18, IL-1β, and TNF-1α was also detected and found to be higher in CCI rats, followed by a significant decrease in CCI mice treated with Exo-NGF (Figs. **4D**-**F**, F* (DFn, DFd) = 202.3 (2.000, 3.059), 122.3 (2.000, 3.559) and 95.04 (2.000, 3.156)). Additionally, Exo-NGF treatment did not significantly change the levels of IL-18, IL-1β, and TNF-1α in the sham rats (Figs. **S1C-S1E**). Therefore, it could be concluded that Exo-NGF treatment could alleviate the inflammatory response of the spinal dorsal horn caused by CCI.

### 3.5 Exo-NGF Inhibited NLRP3 Inflammasome Activation

**Fig. (5):**
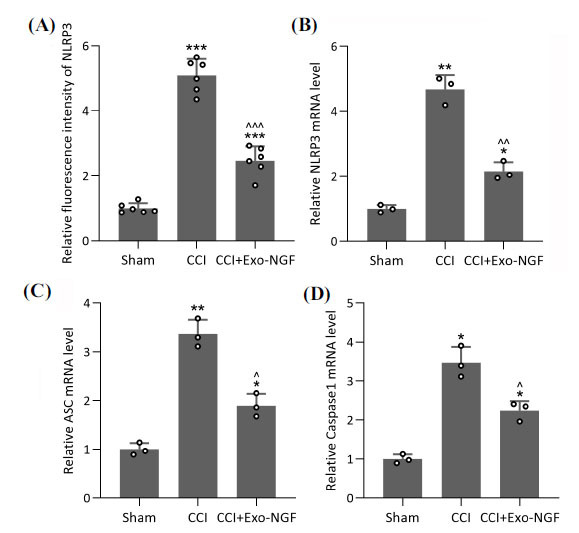
NGF overexpressed exosomes inhibited NLRP3 inflammasome activation in CCI rats. (**A**) was compared. n = 6 for each group. The mRNA expressions of NLRP3 (**B**), ASC (**C**), and Caspase1 (**D**) in the spinal dorsal horn were measured by RT-qPCR. n = 3 repeats for each group (6 tissue homogenates were mixed for each group). Data were presented as mean ± SD. *p < 0.05, **p < 0.01, ***p < 0.001 compared to Sham. ^*p* < 0.05, ^^*p* < 0.01, ^^^*p* < 0.001 compared to CCI group. Brown-Forsythe ANOVA test followed by Dunnett's T3 multiple comparisons test.

Notably, the release of IL-1β and IL-18 was triggered by the NLRP3 inflammasome [27]. Therefore, the effect of Exo-NGF on NLRP3 expression was evaluated. The NLRP3 level in the spinal dorsal horn of rats from different groups was detected using immunostaining. It was found that the intensity of NLRP3 was highest in CCI rats compared to control rats (Fig. **5A**, F* (DFn, DFd) = 160.5 (2.000, 10.83)). However, it was dramatically decreased in CCI rats treated with Exo-NGF (Fig. **5A**). Moreover, the mRNA level of NLRP3 was also highest in CCI rats, followed by a significant decrease in CCI rats when treated with Exo-NGF (Fig. **5B**, F* (DFn, DFd) = 113.0 (2.000, 3.740)).

**Fig. (6):**
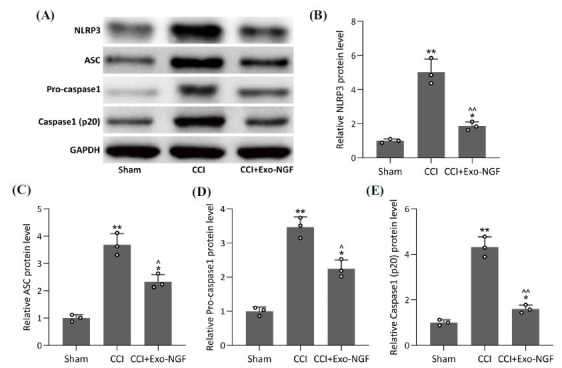
NGF overexpressed exosomes inhibited NLRP3 inflammasome activation in CCI rats. (**A**) Western blot was used to measure the protein level of NLRP3, ASC, Pro-caspase1, and Caspase1 (P20) in the spinal dorsal horn of rats from different groups. GAPDH was used as a loading control, and the expression level was normalized to sham (**B**-**E**). n = 3 repeats for each group (6 tissue homogenates were mixed for each group). Data were presented as mean ± SD. *p < 0.05, **p < 0.01 compared to Sham. ^*p* < 0.05, ^^*p* < 0.01, compared to the CCI group. Brown-Forsythe ANOVA test followed by Dunnett's T3 multiple comparisons test.

The components of NLRP3 inflammasome, including ASC and caspase1, were also detected using qPCR, and the results showed that the levels of ASC and caspase1 mRNA were higher in CCI rats, followed by a drop in CCI rats when treated Exo-NGF (Figs. **5C**, **D**, F* (DFn, DFd) = 79.51 (2.000, 4.622) and 58.47 (2.000, 3.708)). The protein level of NLRP3 was assessed in rats with CCI and rats treated with Exo-NGF. It was observed that NLRP3 levels increased significantly in rats with CCI. However, in rats treated with Exo-NGF, NLRP3 levels decreased noticeably (Figs. **6A**, **B**, F* (DFn, DFd) = 63.01 (2.000, 2.522)). Furthermore, the levels of the other two inflammasome components, ASC, pro-caspase1, and caspase1, were also measured. ASC, pro-caspase1, and caspase1 levels were higher in rats with CCI compared to control rats. Nonetheless, treatment with Exo-NGF resulted in a significant decrease in the levels of these three proteins (Figs. **6C**-**E**, F* (DFn, DFd) = 65.24 (2.000, 3.848), 79.46 (2.000, 4.604) and 115.7 (2.000, 2.937)). Besides, Exo-NGF treatment did not affect the expression of NLRP3 inflammasome-related proteins, including NLRP3, ASC caspase1, and pro-caspase1, in the sham rats (Figs. **S1F-S1J**). These findings clearly demonstrate that exosomes carrying NGF can effectively inhibit the activation of NLRP3 inflammasomes in the spinal dorsal horn induced by CCI.

## 4 DISCUSSION

Neuropathic pain is typically caused by injury or disease of the somatosensory system and often arises following nerve damage. Previous studies have conclusively demonstrated that the administration of mesenchymal stem cell-derived exosomes is a highly effective cell-free therapy for alleviating neuropathic pain [28]. The antinociceptive effects were partially attributed to exosome-mediated interference with Rsad2 expression, leading to the inhibition of microglial activation and the pro-inflammatory response. NGF is one of the most well-known neurotrophic factors, with exceptional potential in treating CNS diseases, as it effectively inhibits pro-inflammatory responses [29]. Therefore, whether NGF-loaded exosomes have a similar effect in alleviating neuropathic pain requires further investigation.

The purified exosomes derived from HEK293 cells were characterized using TEM, and the result revealed that the average diameter of the exosome was about 120 nm. Minnone *et al*. isolated the exosome from human mesenchymal stem cells and found the average diameter was around 70 nm [29], which was smaller than our exosome derived from HEK293 cells in average diameter. However, Yang *et al*. also isolated the exosome from HEK293 cells and found its average diameter was also around 120 nm, which was consistent with our finding [30]. These findings demonstrated that the diameter of the exosome derived from HEK293 cells was larger than that derived from mesenchymal stem cells. Importantly, it is worth noting that Yang *et al*. also successfully loaded the NGF into exosomes derived from HEK293 cells. They explored the effect of Exo-NGF on the alleviation of cerebral ischemia while we focused on neuropathic pain. Our result showed that the NGF protein and mRNA were successfully loaded into exosomes derived from HEK293 cells, with evidence that NGF protein and mRNA levels were strongly higher in Exo-NGF than in exosomes. However, the fold change of NGF mRNA in the Yang *et al*. study was significantly higher than in our study. This difference may be due to the greater amount of NGF plasmid used for transfection in the Yang *et al*. study compared to our study.

When we explored the delivery capacity of Exo-NGF into NSC recipient cells, we found that the NGF mRNA level in NSCs gradually increased with the increasing dose of Exo-NGF treatment. However, Yang *et al*. found that the amount of NGF mRNA in NSCs increased most in the 100 μg dose. The possible reason for this difference is that the amount of NGF mRNA in our Exo-NGF is relatively low, which leads to continuous intake. It has been shown that NGF can promote cell differentiation [31]. Our analysis also revealed that Exo-NGF induced the differentiation of NSCs, as evidenced by the high levels of Tuj-1 and MAP-2. In conclusion, it was found that Exo-NGF can be taken up by NSCs and promote their differentiation. Therefore, the promotion of NSC differentiation could significantly alleviate mechanical allodynia and thermal hyperalgesia in CCI rats.

The reason why male rats are commonly used in pain studies is due to several factors, including historical practices, practical considerations, and scientific biases. A key reason researchers have traditionally favored males is the complexity introduced by the female reproductive cycle, which involves hormonal fluctuations [32]. These fluctuations were thought to introduce unwanted variability in experimental outcomes, especially in pain studies, where hormones can affect pain perception [32]. However, recent research emphasizes the importance of including both male and female animals in neuropathic pain studies. It is now understood that sex hormones can influence pain pathways and responses to analgesics. For example, females may use different immune cell populations (*e.g*., T-cells) to mediate pain responses compared to males, who might rely more on microglia [33-35]. Recent findings from two different neuropathic pain models suggest that certain treatment targets may be more relevant for addressing pain in females [36]. Despite this, using only males simplifies experiments by eliminating a perceived variable, making results easier to reproduce and reducing costs. While convenient, we admit that this approach introduces bias and limits the ability to understand female-specific pain mechanisms.

In the CCI model of neuropathic pain, the choice is usually between analyzing the DRG or the spinal dorsal horn. In our study, we focused on the spinal dorsal horn. The inflammatory response within nerve tissue plays a crucial role in driving the progression of neuropathic pain. Datta-Mitra *et al*. found that NGF significantly upregulates IL-1β at both the protein and mRNA levels in a caspase-1-dependent manner [37]. Furthermore, NGF was observed to induce caspase-1 activation through NLRP1/NLRP3 inflammasomes. Our analysis demonstrated a substantial increase in pro-inflammatory factors, including IL-18, IL-1β, and TNF-α, in the spinal dorsal horn of CCI rats. Remarkably, contrary to previous findings, the injection of Exo-NGF derived from HEK293 cells into the spinal dorsal horn of CCI rats led to a significant reduction in both protein and mRNA levels of these inflammatory factors. The differing findings on the effect of NGF on inflammation are supported by the fact that NGF exhibits both pro-inflammatory and anti-inflammatory roles. However, the mechanism by which NGF suppresses inflammation requires further investigation.

The activation of the NLRP3 inflammasome primarily contributes to inflammatory response, which is of critical importance in neuropathic pain [38]. Cowie *et al*. have demonstrated that male mice lacking NLRP3 exhibit reduced inflammatory infiltration at the surgical site [39]. Consistent with previous studies, we have found that the NLRP3 was activated strongly in CCI rats while decreased in CCI rats treated with Exo-NGF, as evidenced by the immunostaining. However, we only focused on the inflammation in the spinal dorsal horn without distinguishing the different neuronal cell types. Importantly, the primary components of the NLRP3 inflammasome, including ASC and pro-caspase1, also increased in CCI rats. These findings proved that NLRP3 was involved in the neuropathic pain in CCI rats, and Exo-NGF treatment significantly decreased the inflammasome and inflammatory factors, finally alleviating the neuropathic pain in CCI rats.

Although our results have demonstrated the function of Exo-NGF in alleviating neuropathic pain by inhibiting the NLRP3-mediated inflammation response, there are still some limitations in our study. Initially, the mechanism of NGF regulating the activation of NLRP3 was unclear. Moreover, the effect of NGF on alleviating neuropathic pain through NLRP3 requires further experiments to confirm. For example, studying the treatment effect of Exo-NGF on CCI rats with NLRP3 knocking down. Additionally, the pattern of NGF mRNA translation in the NSCs after receiving Exo-NGF must include the 0-h time point. This is crucial to conclusively figure out whether NGF protein in Exo-NGF can be directly delivered into NSCs.

## CONCLUSION

NGF protein and mRNA were successfully loaded into exosomes from NGF-overexpressed HEK293 cells. The level of NGF protein translated from delivered NGF mRNA reached the highest level at 8 h after Exo-NGF treatment in NSCs. Consequently, Exo-NGF promoted the differentiation of NSCs. Moreover, the injection of Exo-NGF into the spinal dorsal horn of CCI rats significantly reduced neuropathic pain. The molecular exploration revealed that Exo-NGF could decrease the NLRP3 inflammasome activation and inflammatory factors induced in CCI rats.

## Data Availability

The data could not be shared openly, as required by our department. The raw data could be obtained upon reasonable request to the corresponding author.

## LISTS OF ABBREVIATIONS

ASC: Adaptor Apoptosis-associated Speck-like Protein
CCI: Chronic Constriction Injury
IL-18: Interleukin-18
IL-1β: Interleukin-1β
MAP-2: Microtubule-associated Protein-2
NGF: Nerve Growth Factor
NLRP3: NOD-like Receptor Family Pyrin Domain-containing Protein 3
NSC: Neuron Stem Cells
TNF-α: Tumor Necrosis Factor α

## SUPPLEMENTARY MATERIAL

Supplementary material is available on the publisher’s website along with the published article.

## References

[1] Cohen S.P., Mao J. (2014). Neuropathic pain: mechanisms and their clinical implications.. BMJ.

[2] Colloca L., Ludman T., Bouhassira D., Baron R., Dickenson A.H., Yarnitsky D., Freeman R., Truini A., Attal N., Finnerup N.B., Eccleston C., Kalso E., Bennett D.L., Dworkin R.H., Raja S.N. (2017). Neuropathic pain.. Nat. Rev. Dis. Primers.

[3] Safieh-Garabedian B., Nomikos M., Saadé N. (2019). Targeting inflammatory components in neuropathic pain: The analgesic effect of thymulin related peptide.. Neurosci. Lett..

[4] Bhagwani A., Chopra M., Kumar H. (2022). Spinal cord injury provoked neuropathic pain and spasticity, and their gabaergic connection.. Neurospine.

[5] Gadot R., Smith D.N., Prablek M., Grochmal J.K., Fuentes A., Ropper A.E. (2022). Established and emerging therapies in acute spinal cord injury.. Neurospine.

[6] Lewis N.E., Tabarestani T.Q., Cellini B.R., Zhang N., Marrotte E.J., Wang H., Laskowitz D.T., Abd-El-Barr M.M., Faw T.D. (2022). Effect of acute physical interventions on pathophysiology and recovery after spinal cord injury: A comprehensive review of the literature.. Neurospine.

[7] Meacham K., Shepherd A., Mohapatra D.P., Haroutounian S. (2017). Neuropathic pain: Central vs. peripheral mechanisms.. Curr. Pain Headache Rep..

[8] Jensen T.S., Finnerup N.B. (2014). Allodynia and hyperalgesia in neuropathic pain: Clinical manifestations and mechanisms.. Lancet Neurol..

[9] Lamkanfi M., Dixit V.M. (2014). Mechanisms and functions of inflammasomes.. Cell.

[10] Hua T., Yang M., Song H., Kong E., Deng M., Li Y., Li J., Liu Z., Fu H., Wang Y., Yuan H. (2022). Huc-MSCs-derived exosomes attenuate inflammatory pain by regulating microglia pyroptosis and autophagy via the miR-146a-5p/TRAF6 axis.. J. Nanobiotechnology.

[11] Shi J., Zhao Y., Wang K., Shi X., Wang Y., Huang H., Zhuang Y., Cai T., Wang F., Shao F. (2015). Cleavage of GSDMD by inflammatory caspases determines pyroptotic cell death.. Nature.

[12] Nakahira M., Nakanishi K. (2011). Requirement of GATA-binding protein 3 for Il13 gene expression in IL-18-stimulated Th1 cells.. Int. Immunol..

[13] Walsh J.G., Reinke S.N., Mamik M.K., McKenzie B.A., Maingat F., Branton W.G., Broadhurst D.I., Power C. (2014). Rapid inflammasome activation in microglia contributes to brain disease in HIV/AIDS.. Retrovirology.

[14] Zheng T., Wang Q., Bian F., Zhao Y., Ma W., Zhang Y., Lu W., Lei P., Zhang L., Hao X., Chen L. (2021). Salidroside alleviates diabetic neuropathic pain through regulation of the AMPK-NLRP3 inflammasome axis.. Toxicol. Appl. Pharmacol..

[15] Cheng K.I., Chen S.L., Hsu J.H., Cheng Y.C., Chang Y.C., Lee C.H., Yeh J.L., Dai Z.K., Wu B.N. (2021). Loganin prevents CXCL12/] CXCR4-regulated neuropathic pain via the NLRP3 inflammasome axis in nerve-injured rats.. Phytomedicine.

[16] Liu W., Wang Y., Gong F., Rong Y., Luo Y., Tang P., Zhou Z., Zhou Z., Xu T., Jiang T., Yang S., Yin G., Chen J., Fan J., Cai W. (2019). Exosomes derived from bone mesenchymal stem cells repair traumatic spinal cord injury by suppressing the activation of A1 neurotoxic reactive astrocytes.. J. Neurotrauma.

[17] Osier N., Motamedi V., Edwards K., Puccio A., Diaz-Arrastia R., Kenney K., Gill J. (2018). Exosomes in acquired neurological disorders: New insights into pathophysiology and treatment.. Mol. Neurobiol..

[18] Keefe K., Sheikh I., Smith G. (2017). Targeting neurotrophins to specific populations of neurons: NGF, BDNF, and NT-3 and their relevance for treatment of spinal cord injury.. Int. J. Mol. Sci..

[19] Han Z., Wang C.P., Cong N., Gu Y.Y., Ma R., Chi F.L. (2017). Therapeutic value of nerve growth factor in promoting neural stem cell survival and differentiation and protecting against neuronal hearing loss.. Mol. Cell. Biochem..

[20] Song Z., Wang Z., Shen J., Xu S., Hu Z. (2017). Nerve growth factor delivery by ultrasound-mediated nanobubble destruction as a treatment for acute spinal cord injury in rats.. Int. J. Nanomedicine.

[21] Wang Y.Q., Wang J., Xia S., Gutstein H.B., Huang Y.H., Schlüter O.M., Cao J.L., Dong Y. (2021). Neuropathic pain generates silent synapses in thalamic projection to anterior cingulate cortex.. Pain.

[22] Sun K., Zhang H., Zhang T., Sun N., Hao J., Wang Z., Gao C. (2023). Spinal HDAC6 mediates nociceptive behaviors induced by chronic constriction injury via neuronal activation and neuroinflammation.. Mol. Pain.

[23] Chen S.H., Lin Y.W., Tseng W.L., Lin W.T., Lin S.C., Hsueh Y.Y. (2024). Ultrahigh frequency transcutaneous electrical nerve stimulation for neuropathic pain alleviation and neuromodulation.. Neurotherapeutics.

[24] Xue C., Kui W., Huang A., Li Y., Li L., Gu Z., Xie L., Kong S., Yu J., Ruan H., Wang K. (2024). Electroacupuncture suppresses neuronal ferroptosis to relieve chronic neuropathic pain.. J. Cell. Mol. Med..

[25] Fairbanks C.A. (2003). Spinal delivery of analgesics in experimental models of pain and analgesia.. Adv. Drug Deliv. Rev..

[26] Wang J., Sun H., Guo R., Guo J., Tian X., Wang J., Sun S., Han Y., Wang Y. (2023). Exosomal miR-23b-3p from bone mesenchymal stem cells alleviates experimental autoimmune encephalomyelitis by inhibiting microglial pyroptosis.. Exp. Neurol..

[27] Chen C., Smith M.T. (2023). The NLRP3 inflammasome: Role in the pathobiology of chronic pain.. Inflammopharmacology.

[28] Shiue S.J., Rau R.H., Shiue H.S., Hung Y.W., Li Z.X., Yang K.D., Cheng J.K. (2019). Mesenchymal stem cell exosomes as a cell-free therapy for nerve injury-induced pain in rats.. Pain.

[29] Minnone G., Benedetti D.F., Bracci-Laudiero L. (2017). NGF and its receptors in the regulation of inflammatory response.. Int. J. Mol. Sci..

[30] Yang J., Wu S., Hou L., Zhu D., Yin S., Yang G., Wang Y. (2020). Therapeutic effects of simultaneous delivery of nerve growth factor mRNA and protein via exosomes on cerebral ischemia.. Mol. Ther. Nucleic Acids.

[31] Chung J., Kubota H., Ozaki Y., Uda S., Kuroda S. (2010). Timing-dependent actions of NGF required for cell differentiation.. PLoS One.

[32] Mogil J.S., Chanda M.L. (2005). The case for the inclusion of female subjects in basic science studies of pain.. Pain.

[33] Coraggio V., Guida F., Boccella S., Scafuro M., Paino S., Romano D., Maione S., Luongo L. (2018). Neuroimmune-driven neuropathic pain establishment: A focus on gender differences.. Int. J. Mol. Sci..

[34] Sorge R.E., Mapplebeck J.C.S., Rosen S., Beggs S., Taves S., Alexander J.K., Martin L.J., Austin J.S., Sotocinal S.G., Chen D., Yang M., Shi X.Q., Huang H., Pillon N.J., Bilan P.J., Tu Y., Klip A., Ji R.R., Zhang J., Salter M.W., Mogil J.S. (2015). Different immune cells mediate mechanical pain hypersensitivity in male and female mice.. Nat. Neurosci..

[35] Sorge R.E., Totsch S.K. (2017). Sex differences in pain.. J. Neurosci. Res..

[36] Boccella S., Guida F., Logu D.F., Gregorio D.D., Mazzitelli M., Belardo C., Iannotta M., Serra N., Nassini R., Novellis d.V., Geppetti P., Maione S., Luongo L. (2019). Ketones and pain: Unexplored role of hydroxyl carboxylic acid receptor type 2 in the pathophysiology of neuropathic pain.. FASEB J..

[37] Datta-Mitra A., Kundu-Raychaudhuri S., Mitra A., Raychaudhuri S.P. (2015). Cross talk between neuroregulatory molecule and monocyte: Nerve growth factor activates the inflammasome.. PLoS One.

[38] Wang Y., Li Y., Wang J., Zhao Q., Wen S., Wang S., Sun T. (2020). A novel mechanism of specialized proresolving lipid mediators mitigating radicular pain: The negative interaction with NLRP3 inflammasome.. Neurochem. Res..

[39] Cowie A.M., Menzel A.D., O’Hara C., Lawlor M.W., Stucky C.L. (2019). NOD-like receptor protein 3 inflammasome drives postoperative mechanical pain in a sex-dependent manner.. Pain.

